# Estrogen Receptors as Key Factors in Carcinogenesis

**DOI:** 10.3390/biomedicines13112620

**Published:** 2025-10-26

**Authors:** Oliwia Gruszka, Magdalena Jurzak, Ilona Anna Bednarek

**Affiliations:** Department of Biotechnology and Genetic Engineering, Faculty of Pharmaceutical Sciences in Sosnowiec, Medical University of Silesia, 40-055 Katowice, Poland; magda@sum.edu.pl

**Keywords:** estrogen receptor, tumour microenvironment, breast cancer, ovarian cancer, thyroid cancer, colorectal cancer, cancer therapy

## Abstract

Despite continuous advances in the development of methodologies for the diagnosis and therapeutic treatment of cancer, the disease remains a primary cause of mortality worldwide. A comprehensive understanding of the molecular mechanisms underlying cancer could ultimately lead to increasingly effective therapeutic interventions. One approach that could be adopted is to formulate methodologies that impede cell signalling and/or the expression of genes pivotal to carcinogenesis. A notable example of this strategy is the focus on the estrogen receptor, a key player in the development of various types of cancer. The deregulation of this receptor, and the subsequent impact on cell function, is a critical factor in the progression of these diseases. This renders it a significant therapeutic target. Furthermore, the microenvironment has been demonstrated to exert a significant influence on the development of cancers. A mounting body of evidence indicates that the abnormal physical properties of the tumour microenvironment can induce widespread changes, leading to the selection of characteristic tumour cell abilities and subsequent clonal proliferation. This process is accompanied by an increased capacity for invasive growth and, notably, the induction of multidrug resistance. The present article focuses on presenting the structure and role of the estrogen receptor in selected hormone-dependent cancers, its involvement in the formation of the tumor microenvironment, currently used therapeutic methods in the treatment of these cancers, and the challenges associated with them. Each new discovery in the field of cancer biology offers the prospect of developing new potential treatments, including targeted therapies aimed at improving the survival of patients suffering from hormone-dependent malignant tumours. Although the role of the estrogen receptor in their development is well established, further research is required to develop a detailed understanding of how its specific isoforms act in different types of cancer.

## 1. Introduction

On a global scale, cancer is considered to be one of the most significant health and economic challenges of the 21st century. Research has shown that cancer accounts for around one-third of premature deaths [1]. Prospective analyses indicate that, by 2040, there will be nearly 40 million new cancer cases, with mortality exceeding 15 million [2]. This will have a profound impact on millions of individuals worldwide, contributing significantly to cancer-related morbidity and mortality. A detailed analysis of cancer cases has revealed significant gender disparities in the incidence of certain types of cancer. Research has shown that this is due to hormones secreted by the gonads [3].

Human hormones are specialised chemical compounds that are produced by endocrine cells with the specific function of regulating the activity of various types of cells. These hormones are responsible for regulating numerous physiological functions within the human body. The levels of these hormones fluctuate depending on the developmental stage of the body. The balance of these elements is crucial for the optimal functioning of the body as a whole [4]. Disorders in homeostatic mechanisms can lead to adverse health consequences, manifesting as metabolic diseases, including obesity, diabetes, cardiovascular disease and cancer [5]. The role of these cells in the process of carcinogenesis is primarily to stimulate specific receptors. The activation of these receptors leads to a cascade of cellular events, the end result of which is the regulation of gene expression. The process of carcinogenesis is initiated by the activation of genes responsible for promoting cell growth and proliferation and/or inhibiting apoptosis and stimulating angiogenesis [6]. Sex hormones have been identified as playing a pivotal role in some cancers’ development [7]. These cancers are classified into a separate group referred to as hormone-dependent cancers [8]. Evidently, both androgen and estrogen hormones play a pivotal role in the initiation, development and progression of hormone-dependent cancers. This may be due to increased hormone concentrations, receptor overexpression, or constitutive stimulation caused by mutation [9]. The important role of estrogen receptors (ERs) in physiological and biological processes such as growth, development and metabolism has been demonstrated [10]. Stimulating ERs has been shown to activate transcriptional processes and signalling events, resulting in the regulation of gene expression [11,12]. This aspect of ER function is also associated with their involvement in pathological processes [12]. There is a demonstrated correlation between ER activity and various types of cancer, including prostate, breast, ovarian, endometrial and lung cancer [10]. In addition to the well-established classical molecular pathways, the estrogen receptor has also been shown to interact with the tumor microenvironment, driving carcinogenesis further [13]. Furthermore, numerous reports have emerged regarding their involvement in neurodegenerative diseases, coronary diseases, osteoporosis [10], gastrointestinal diseases and even melanoma [14].

Several forms of estrogen receptors have been described. Among these are the estrogen receptor alpha and beta, which have numerous isoforms, and the estrogen receptor coupled with G protein. The estrogen receptor alpha (ERα) was first described in the 1950s. For several years, it was considered the only receptor protein, until 1996, when the estrogen receptor beta (ERβ) was discovered, exhibiting similar properties. The most recent discovery was the G protein-coupled estrogen receptor class A (GPER) [15].

In the context of hormone-dependent cancers, estrogen receptors play a particularly important role in the tumour microenvironment (TME). The components of the tumour interact with each other to influence the development and progression of cancer. It is evident that both cancer cells and the surrounding normal cells, including immune system cells, participate in the formation of the TME. Another significant component of the TME is the extracellular matrix (ECM), which consists of active tissue components that facilitate intercellular communication, proliferation and adhesion [16]. The expression of estrogen receptors has been observed in the cells that constitute the TME. These cells influence the TME by activating pathways that stimulate microenvironment components [17].

Understanding the mechanisms by which the estrogen receptor is activated in pathways involved in cancer development has rendered ER a very promising therapeutic target [18]. Consequently, numerous therapeutic interventions have been developed for cancers that express ER. Two examples of anti-estrogens that have been developed are selective estrogen receptor modulators (SERMs) and selective estrogen receptor downregulators (SERDs). These are designed to compete with estrogen for binding to ER. The mechanism of action of selective estrogen receptor modulators involves a change in the configuration of the receptor, thereby preventing the formation of an active complex. Conversely, SERD activity ultimately results in ER degradation. In addition, there are other groups of drugs that have been demonstrated to affect gene activation by ER. For instance, aromatase inhibitors (AIs) implicated in estradiol synthesis impose constraints on the production of the ligand indispensable for ER activation, consequently engendering a diminution in its activity [19]. However, it has been demonstrated that the activity of some of these genes can be negated or reduced by a mutation in the gene encoding ER [20]. Furthermore, it has been hypothesised that certain components of the TME, notably immune cells, may facilitate the development of drug resistance in cancer cells [21]. These limitations, primarily associated with SERMs and AIs, may be surmounted by the emergence of SERD-based therapies [22]. Therapies based on the use of cyclin-dependent kinase 4/6 inhibitor (CDK4/6i) have been developed, which are insensitive to resistance associated with mutations in the ER gene and result in prolonged progression-free survival in patients with advanced breast cancer [23].

## 2. Structure and Signaling of the Estrogen Receptor

**Figure 1:**
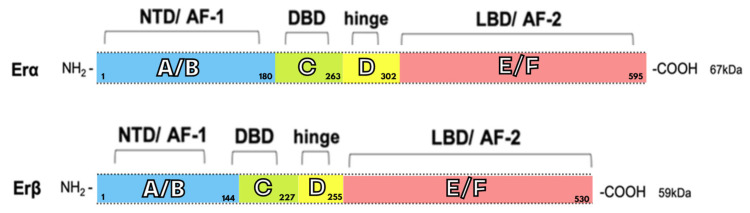
The structural organisation of alpha and beta estrogen receptors. NTD: gene transcription mechanism; DBD: DNA-binding domain; LBD: ligand-binding domain; AF-1: activation function 1; AF-2: activation function 2.

It is evident that estrogen receptors alpha and beta are classified as members of the superfamily of nuclear receptors. The analysis revealed that they exhibit common structural features in several regions. However, their functional domains demonstrate significant differences. ERα consists of 595 amino acids and has a molecular weight of 67 kDa. Conversely, ERβ comprises 530 amino acids and has a molecular weight of 59 kD (Figure 1). This variation between the two proteins is due to the shorter amino domain present in the beta receptor. In humans, the alpha receptor is encoded by the *ESR1* (estrogen receptor 1) gene. Its location on chromosome 6 is at locus 6q25.1 [18,24].

The letters A/B, C, D and E/F are assigned to the basic functional domains. Region A/B corresponds to the N-terminal nucleotide binding domain (NTD), which is responsible for transactivation with the gene transcription mechanism. This fragment has been found to contain zinc fingers, which are known to mediate binding to target sequences. The subsequent region, marked with the letter C, is responsible for the DNA-binding domain (DBD). It contributes to the process of dimerisation of the estrogen receptor and to the process of binding to specific sequences in chromosomes. Domain D has been identified as a hinge region, functioning as a flexible link between domains C and E. It has been demonstrated to bind to chaperone proteins and contains a nuclear localisation signal that is exposed following estrogen binding. Consequently, the receptor-ligand complex is able to enter the cell nucleus. The carboxylic terminus of the protein, designated as region E/F, has been identified as the ligand-binding domain (LBD). This region has been found to contain the estrogen binding site, in addition to binding sites for coactivators and corepressors. AF1 and AF2, located in the NTD and DBD, respectively, are two additional transcriptional activity regulators known as activation function (AF) domains [14,18,25,26].

In addition to the full-length (67 kDa) form of ERα, several isoforms have been identified that differ in length [18]. Exemplary isoforms in this category include ERα46 and ERα36, whose presence is associated with the development and progression of cancers such as breast and thyroid cancer. Some of these genes are unable to activate transcription because they lack the NTD region, and thus the functional AF-1 domain. However, the observed binding of these molecules to the full-length ERα receptor results in the formation of heterodimers, thereby inhibiting the ability of the receptor to regulate transcription. In turn, the smallest variant, ERα-36, does not contain transcription-activating domains (activation function 1, AF1, and activation function 2, AF2) in its structure [24].

The estrogen receptor beta is encoded by the *ESR2* (estrogen receptor 2) gene, which is located on chromosome 14 at locus 14q23-24. The protein is classified as a nuclear receptor and is predominantly located in the cell nucleus; however, evidence suggests that it is also present in the cytoplasm and mitochondria. To date, no link has been established between cellular location and ERβ function [18]. The beta estrogen receptor occurs in five different variants. These are distinguished as ERβ1 to ERβ5, and are the result of alternative splicing. However, it is only full-length beta receptors that are capable of binding antagonistic and agonistic ligands. Furthermore, shorter forms of ERβ are unable to form homodimers and, in addition, have no intrinsic activity of their own. Conversely, they have the potential to heterodimerise with ERβ and even with ERα, leading to the induction of transcriptional activity in a ligand-dependent manner. This is particularly important in the study of the interaction and mutual regulation of related receptors [27].

A recently identified atypical estrogen receptor has been found to be coupled with a class A G protein. This G protein-coupled estrogen receptor has been identified as a membrane estrogen receptor that is not associated with the nuclear ER. It is located in the cytoplasmic membrane and has the ability to move to the endoplasmic reticulum membrane. The gene that encodes GPER is located on chromosome 7 (locus 7p22.3) [28]. Its structural configuration deviates from the two fundamental types of ERs. This receptor’s structure is typical of a G protein-coupled receptor, consisting of seven transmembrane α-helical regions, four extracellular segments, and four cytosolic segments [29]. It is evident that, owing to its divergent structural characteristics, GPER functions via an alternative mechanism to conventional nuclear receptors, such as ER α and β. The role of GPER as an estrogen mediator has been demonstrated in various physiological systems, including the nervous, immune, and cardiovascular systems. Furthermore, it has been shown to play a pivotal role in the metabolic processes of bones, kidneys and the pancreas [28,30], The structure of this receptor results in a comparatively low level of affinity for estradiol binding in comparison to other hormone receptors. However, this becomes an advantage for GPER, as it functions as a secondary messenger in rapid responses to estrogen and the activation of intracellular signalling cascades [18].

ER signalling can be initiated via either genomic or non-genomic pathways. Genomic signalling can be categorised into two distinct types: direct and indirect. The study is based on the premise that ligand-activated ER impacts DNA. In the case of direct genomic signalling, the dimerised ERα or ERβ complex is transported from the cytoplasm to the cell nucleus, where it interacts directly with the estrogen response elements sequence (EREs) within gene promoters, thereby initiating their expression. In contrast, indirect genomic signalling relies on interactions between secondary signal transducers and gene expression, which are initiated by ER activation via a ligand. The non-genomic pathway, in turn, involves the influence of activated ER on intracellular signalling pathways that initiate gene expression [18].

**Figure 2:**
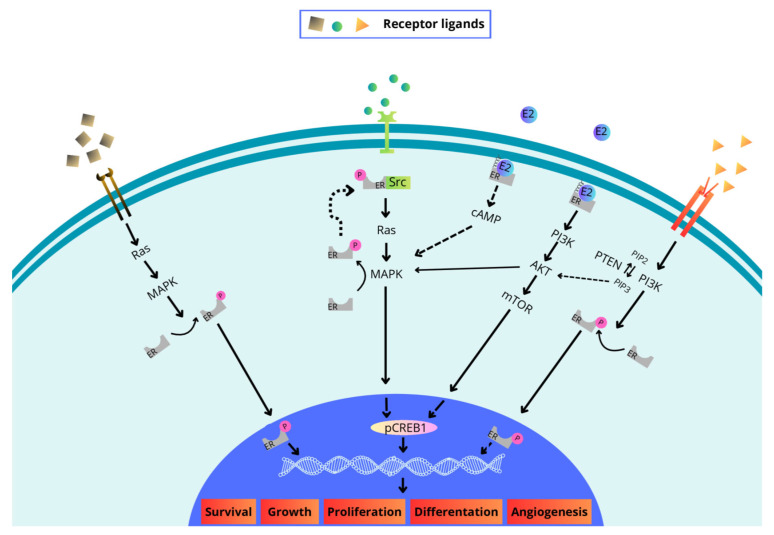
The role of the ER in the activation of various signaling pathways in cells. ER: estrogen receptor; Ras: GTPase HRas; MAPK: mitogen-activated protein kinase; P: phosphorylated form; Src: proto-oncogene tyrosine-protein kinase; pCREB1: phosphorylated cAMP-responsive element-binding protein 1; cAMP: cyclic adenosine monophosphate; PI3K: phosphatidylinositol 3-kinase; AKT: protein kinase B; mTOR: mammalian target of rapamycin; PTEN: phosphatidylinositol-3,4,5-trisphosphate 3-phosphatase; PIP2: phosphatidylinositol 4,5-bisphosphate; PIP3: phosphatidylinositol 3,4,5-trisphosphate.

Activation of estrogen receptors has been shown to result in the expression of genes that regulate processes such as proliferation and cell differentiation, apoptosis and autophagy, and vasodilation (Figure 2). Consequently, imbalances in the quantity and activity of these receptors have been linked to numerous types of cancer [14].

## 3. Role of TME

A comprehensive understanding of the intricate interactions between the components of the tumour microenvironment is imperative for the development of future effective cancer therapies. To this end, the following presentation will summarise the latest reports on TME in hormone-dependent cancers.

Cancer-associated fibroblasts (CAFs) appear to play a key role in the tumour microenvironment. These cells are classified into multiple subpopulations, which have been observed to be associated with an increased risk of tumour development through various mechanisms. To illustrate this point, in the context of colorectal cancer (CRC), the presence of cancer-associated fibroblasts that express the melanoma cell adhesion molecule (MCAM) has been shown to correlate with a more aggressive clinical course and a poorer prognosis for patients [31]. Despite the presence of differences in subpopulations, the majority of these cells produce interleukin 6 (IL-6), a key factor in the development of immune resistance in breast cancer (BC) patients [32] and the promotion of thyroid cancer (TC) cell proliferation [33]. Its inhibition has been demonstrated to result in a weakening of interactions with the STAT3/Akt-PD-L1 pathway, thereby reversing this implication [32]. In a similar manner, CAFs have been observed to exert an influence on other TME cells. Research conducted using a mouse BC model has demonstrated elevated levels of interleukin-2 (IL-2) and interleukin-7 (IL-7), along with cytotoxic T cells and dendritic cells exhibiting antitumour activity, subsequent to the elimination of CAFs. Furthermore, a reduction in tumour-associated macrophages (TAMs), regulatory T lymphocytes (Tregs), vascular endothelial growth factor (VEGF), interleukin-4 (IL-4) and interleukin-6 (IL-6), which have immunosuppressive effects, was observed in BC [34]. Zhang et al. [35] conducted an analysis that identified seven subtypes of TAMs in primary BC samples. A significant proportion of these exhibited pro-tumour characteristics, including influencing angiogenesis via endothelial cells, maintaining inflammation, regulating Tregs and inhibiting T cell function. However, the remaining subtypes exhibited a dual or suppressive effect on BC development [35]. The recruitment of TAMs by CAFs has been linked to the promotion of programmed cell death protein-1 (PD-1) expression [36]. Conversely, the impact of TAMs on the suppression of T cell function via their influence on the programmed cell death ligand 1 (PD-L1) pathway in ovarian cancer (OC) signifies the multifaceted nature of these interactions [37]. Studies of OC have shown that TAMs are involved in forming heterotypic spheroids with high ITGA5 (integrin α5) expression in metastasis [38]. Regarding TC, studies have shown that CAFs are the only risk factor for lymph node metastasis [39]. Research conducted on TC has demonstrated that transforming growth factor beta 1 (TGFβ1), a multifaceted cytokine secreted by tumour cells, directly contributes to the influx of TAMs [40]. Additionally, a correlation has been identified between reduced autophagy in BC and resistance to tamoxifen treatment [41].

The extant research indicates that TAMs contribute to tamoxifen resistance through their activation of the PI3K/Akt/mTOR pathway [42,43]. A study conducted by Qin et al. indicates a mechanism mediated by increased cyclooxygenase-2 (COX-2) expression in TAMs [42], while Li et al. attribute this relationship to CC-chemokine ligand 2 (CCL2) [43]. Furthermore, CCL2 overexpression has been confirmed in TC [44]. The process of epithelial–mesenchymal transition (EMT), which has been demonstrated to promote BC growth and metastasis, has also been associated with tamoxifen resistance. Notch1 and Notch4 have been identified as playing a pivotal role in this process. However, Notch4 exerts a more pronounced effect on the reduction in the effectiveness of tamoxifen in BC by enhancing signal transducer and activator of transcription 3 (STAT3) [45]. EMT has also been linked to cancer stem cells (CSCs) that overexpress CD44 [46]. It is a transmembrane glycoprotein with numerous isomeric variants, which, by interacting with hyaluronic acid (HA), can enhance various further signalling pathways. It has been demonstrated that the adhesive cascade of CRC cells plays a pivotal role in metastasis [47]. Furthermore, studies indicate the expression of hyaluronan synthase 2 (HAS2) by CAFs, which drives the EMT process [48]. The interaction between CD44 and HA may therefore be a potential therapeutic target. The formation of disulphide bonds between cysteine residues is a crucial aspect of the binding of these molecules. Research has identified a particularly significant influence of the bond between Cys77 and Cys92 on cancer cell metastasis. The destabilisation of this bond through redox reactions has been shown to contribute to the therapeutic regulation of the interaction between CD44 and HA [49]. Conversely, the correlation between HA turnover in the ECM and ERα expression highlights the important role of oestrogens in the TME [50]. Advancing knowledge about epigenetic regulation of the TME has the potential to play a substantial role in the development of new treatment methods. In a study on BC, the effect of histone deacetylase 6 (HDAC6) immunosuppression in CAFs was determined by STAT3 activation and increased COX2 expression. In addition, the inhibition of its activity has been shown to reduce the number of M2 subpopulation TAMs, as well as to decrease the recruitment of myeloid-derived suppressor cells (MDSCs) and Tregs [51]. The role of collagen in the TME in cancer is also a very important factor determining the risk of malignant transformation, increased invasiveness and accelerated migration. It has been demonstrated that collagen-1, the protein responsible for increasing the ECMs density, assumes a pivotal role in these processes [52]. The ratio of different collagens is also an important factor in the development of BC. For instance, the suppression of type IV alpha 2 collagen has been demonstrated to inhibit the proliferation and migration of triple-negative breast cancer (TNBC) cells [53].

## 4. Role of ER in TME

A significant proportion of cancers have been observed to display various isoforms of the estrogen receptor [14]. As previously stated, ERs regulate gene expression via multiple signalling pathways. These include cyclic adenosine monophosphate (cAMP), mitogen-activated protein kinase (MAPK), phosphatidylinositol 3-kinase (PI3K), protein kinase B (AKT) [54,55] and Janus kinase (JAK) [56] (Figure 2). This phenomenon is consistent with the conditions created by the tumour microenvironment [57]. The TME comprises a combination of cellular and non-cellular components, including CAFs, endothelial and lymphatic cells, TAMs, tumour-associated neutrophils (TANs), Tregs, and natural killer cells (NK) [58], ECM components, and chemokines [59]. The influence of these components on the pathological development of the disease has been well documented [60]. The cells that compose the TME may contribute to disease progression by expressing and secreting factors that mediate proliferation. The suppression of the anti-tumour immune response or the induction of adaptive immunity has been demonstrated to negate its inhibitory effect on tumour development [61].

CAFs are considered to be one of the key elements of the TME. The influence of these cells on the growth, invasion, metastasis, and drug resistance of cancer cells is a result of their interaction with the cells, as well as with components of the microenvironment [55]. It has been suggested that there is a correlation between CAFs and oestrogen receptor signalling due to the function performed by CAFs [62]. The correlation is complex. A notable aspect of these interactions is revealed by the mutual activation of signalling pathways at multiple levels. For example, studies have shown that estrogen-induced transcription of IL-11 (Interleukin-11), which is, among other things, a molecule of the JAK pathway, significantly increases the migration and invasion induction abilities of CAFs [63]. Additionally, the presence of estrogen receptors in CAFs has been documented. In the context of CAFs derived from TNBC, the expression levels of the ERα36 isoform have been found to fluctuate. Cells with heightened receptor expression demonstrated an escalated production of cytokines and growth factors (e.g., interleukin-1 beta (IL-1β), hepatocyte growth factor (HGF), interleukin-8 (IL-8), VEGF), which were found to provide notable support to tumour progression [64]. In TNBC, a positive correlation was also detected between GPER activity in CAFs and their increased synthesis and secretion of glutamine into the extracellular matrix, accompanied by an increase in tumour cell absorption and metabolic utilisation of glutamine. This had a significant impact on the malignancy of the tumour under study [65]. A study investigating the impact of ERα expression in CAFs in gastric cancer confirmed increased expression of matrix metalloproteinase-2 (MMP2) and matrix metalloproteinase-9 (MMP9). These are responsible for the remodelling of the extracellular matrix, supporting tumour invasion and metastasis [62]. Notably, there are documented accounts of the role of CAFs in regulating the expression of aromatase, a key enzyme in the conversion of androgens into estrogens by cancer cells [55]. The findings of the current studies reveal the pivotal function of the estrogen receptor in tumour progression and its interaction with CAFs. Nevertheless, these findings suggest the possibility of new therapeutic treatments being developed [65].

Pro-tumour immune cells that are part of the tumor immune microenvironment (TIME), such as Tregs and TAMs, play an important role in weakening the anti-tumour immune response and creating an immunosuppressive microenvironment [66]. TAMs can be categorised into two distinct subpopulations: classically activated M1, which exhibits anti-tumour properties, and alternatively activated M2, which demonstrates pro-tumour capabilities. M2-type TAMs primarily function by secreting various growth factors, including VEGF, PDGF, EGF, as well as MMP2 and MMP9, which are metalloproteinases found in the extracellular matrix. In addition, these cells secrete cytokines such as tumour necrosis factor alpha (TNF-α), IL-1β, and IL-8. The net effect of these secretions is to promote processes such as angiogenesis and metastasis. It has also been demonstrated that they secrete heme oxygenase-1 (HO-1) and COX-2, which are involved in the processes of angiogenesis and carcinogenesis [67]. In vitro studies have confirmed that TAMs can contribute to the development of endocrine resistance in estrogen receptor-positive (ER+) breast cancer cells by prolonging the release of TNF-α and interleukin 6 both by themselves and by the cancer cells they affect. This, in turn, resulted in the activation of nuclear factor kappa-light-chain-enhancer (NF-κB), STAT3, extracellular signal-regulated kinase 1 (ERK-1) and constitutively activated ERα through its hyperphosphorylation [68]. In the case of Tregs, these cells demonstrate a relative insensitivity to changes in the extracellular environment due to their epigenetic mode of functional regulation. Their primary function is evidently to regulate autoimmune diseases. Tregs are categorised into subsets based on the type of cytokines they secrete. However, it is important to note that all subsets contribute significantly to the development of immune tolerance in tumours [69]. Tregs are modulated by estrogens. The ERα response element, located in the forkhead box P3 (*FOXP3*) promoter region, is an important transcriptional factor for the immunosuppressive activity of Tregs, and controls its expression [70]. Research has shown that Treg numbers decrease in ER-positive (estrogen receptor-positive) ovarian and breast tumours in mice in response to anti-estrogen treatment. This finding indicates that the administration of anti-estrogenic agents to patients diagnosed with ER+ breast cancer may lead to a reduction in Treg influx and activity, consequently enhancing anti-tumour immune responses [71]. Another type of cell that is part of the TME and is key to the immune suppression of tumours are MDSCs. These cells constitute a heterogeneous population of myeloid cells that accumulate in the TME, where they inhibit the antitumour activity of NK cells and T lymphocytes, among other things [72]. The estrogen receptor exerts a significant influence on the accumulation of MDSC, a process that is facilitated by the expression of STAT3. It has been demonstrated that ER enhances the immunosuppressive function of MDSCs [73]. A multitude of studies have confirmed that an increase in both exogenous and endogenous estrogen concentrations increases the influx of MDSCs and enhances their immunosuppressive effects through ERα activation. This progression has been identified as a contributing factor in the development of cervical cancer [74].

The ECM, which consists of a wide variety of components including proteoglycans/glycosaminoglycans, collagens, laminin, fibronectin, macromolecules, enzymes and proteins, plays a pivotal role in the TME. The ECM is responsible for the transmission of signals between cells and provides a structural framework that facilitates cell adhesion, proliferation, differentiation and migration. It is evident that ER exerts a substantial influence on ECM remodelling, consequently leading to tumour growth and metastasis [75]. Literature reports indicate that matrix metalloproteinases (MMPs) and their inhibitors, tissue inhibitors of metalloproteinases (TIMPs), play a role in cancer cell migration. Research has demonstrated that this process is modulated by the ERα-36-STAT3 complex, which has been observed to bind to the promoters of MMP2 and MMP9 metalloproteinases, function as an enhanceosome complex, and thereby enhance their expression [76].

The ECM has been shown to play a significant role in the development of drug resistance in hormone-dependent tumours. A comprehensive review of the extant literature reveals that studies have demonstrated the involvement of insulin-like growth factor (IGF) activated ERα phosphorylation, which affects the secretion of MMP2/MMP9, which contribute to the development of resistance mechanisms to anti-estrogen therapies through ECM reorganisation [77]. In addition, collagen-1-rich ECM can alter hormonal signals in ERα-positive breast cancer, causing its development and increasing the frequency of lung metastases. An increase in collagen-1 has been shown to shift the activity of signalling cascades in tumour epithelium from signal transducer and activator of transcription 5 (STAT5), a prolactin mediator associated with a milder course of the tumour, towards ERK1/2 and AKT pathways, which are poor prognostic markers [78].

**Figure 3:**
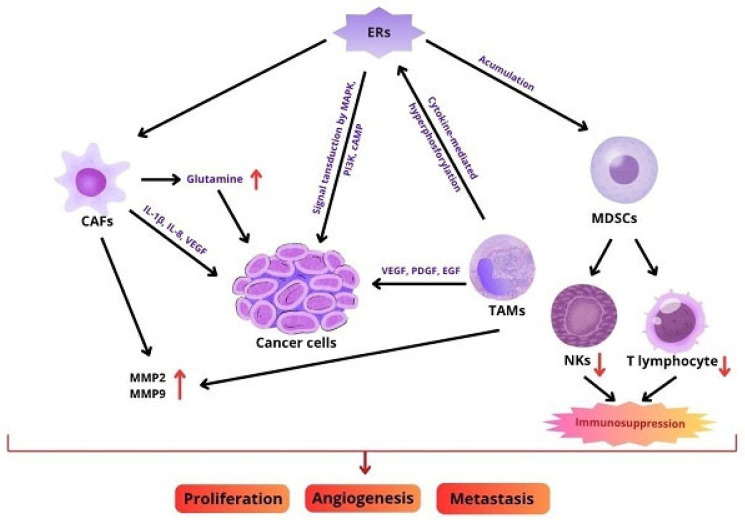
A schematic diagram illustrating ER-mediated signaling in the TME. ERs: estrogen receptors; CAFs: cancer-associated fibroblasts; IL-1β: interleukin-1 beta; IL-8: IL-1β: interleukin-8; VEGF: vascular endothelial growth factor; PDGF: platelet-derived growth factor; EGF: epidermal growth factor; MAPK: mitogen-activated protein kinase; PI3K: phosphatidylinositol 3-kinase; cAMP: cyclic adenosine monophosphate; MDSCs: myeloid-derived suppressor cells; TAMs: tumour-associated macrophages; NKs: natural killer cells; MMP2: matrix metalloproteinase-2; MMP9: Matrix metalloproteinase-9; ↑: increase; ↓: decrease.

It is apparent that interactions between cancer cells, stromal cells, immune cells and extracellular molecules in the tumour microenvironment are significant for carcinogenesis and metastasis. Figure 3 shows the schematic involvement of estrogens/ER in interaction with the TME.

## 5. The Role of the ERs in Specific Cancers

Hormones, particularly steroids, play a key role in the development of tumours or carcinogenesis in various target tissues by influencing their physiological functions and proliferation. In the tumour microenvironment, receptors that respond to signals from the extracellular matrix play a pivotal role in tumour development. In the context of the estrogen receptor, cancers such as ovarian and breast cancer are particularly notable. However, due to their high incidence levels and coverage in the literature, this review will also include thyroid and colon cancers. Below, we present an overview of selected cancers in terms of the presence and activity of the estrogen receptor.

### 5.1. Ovarian Cancer

Ovarian cancer is the principal gynaecological cancer. Its incidence is constantly increasing. In terms of mortality, it is one of the lethally cancers. In 2022, there were 324,603 new cases and over 206,956 deaths [79]. Moreover, it has been determined that half of all OC cases are diagnosed in individuals aged over 65. In the USA, the average age of onset of OC is 57–59 years. However, it is important to note that only 10–15% of cases occur in patients before menopause, which occurs around the age of 51 [80]. In view of these correlations, research has been undertaken to determine the causes of the increased incidence of OC in postmenopausal women. Despite the absence of a definitive understanding of the etiology of ovarian cancer due to its multifaceted nature, several hypotheses have been postulated to explain the origin of OC in relation to hormone levels. Furthermore, the literature describes gonadotropic signalling, the direct action of progesterone and androgens, and ovulation in the progression of this type of cancer [81]. Studies have shown that 17β-estradiol (E2) can stimulate the proliferation of ovarian cells, but it can also induce neoplasia. The estrogen receptor α is present in both normal ovarian cells and cancer cells. OC is characterised based on ERα expression. Increased expression of ERα occurs in over 60% of ovarian cancer cases [82]. ERα is responsible for metastasis and OC progression through specific pathways involving VEGF and MAPK [83]. Nevertheless, this receptor has been strongly associated with improved survival in endometrioid ovarian cancer [15]. Thanks to its presence in OC, many therapeutic strategies have been developed [84].

In addition to ERα, ERβ is also present in ovarian cancer. A study by Bogush et al. confirmed the presence of both receptor forms in OC [85]. In addition to the full-length ERβ, its isoforms are also present [86]. In a normal ovary, ERβ is more abundant than ERα. However, in the case of tumour development, this ratio is reversed, with ERα mRNA levels remaining comparable to those in normal tissue [87]. This correlates with reports of the suppressive effect of ERβ on OC in both in vitro and in vivo studies [15]. Furthermore, studies involving an increase in ERβ levels or its stimulation have resulted in decreased cell motility and proliferation by reducing *MMP2* and cyclin A2 (*CCNA2*) expression and inducing p21 and fibulin-1c ( *FBLN-1C*) expression [88].

In the case of GPER, the rapid and independent intracellular response to extracellular ligands via signal transduction pathways is a negative prognostic factor for the overall survival of ovarian cancer patients [89]. GPER expression levels are relatively low compared to those of ERα and ERβ [28]. However, it correlates with *MMP9* expression, influencing the initiation and migration of OC cells [90]. Nevertheless, some studies have suggested an inhibitory effect of GPER on OC cell proliferation [91]. This may be due, among other things, to the epigenetic effect of GPER on H3K4me3 (Histone H3 lysine 4 trimethylation) [92].

### 5.2. Breast Cancer

Breast cancer is the most commonly diagnosed cancer among the global female population and represents the leading cause of mortality among women with cancer [79]. There is a great amount of evidence that links estrogen to breast cancer. Current evidence appears to confirm estrogen’s oncogenic and tumour-promoting role in breast cancer risk, recurrence and progression. However, a more detailed analysis reveals that the relationship between ostrogen and breast cancer is complex. BC is a group of diseases that present with different molecular profiles and clinical-pathological characteristics. Classification of the different molecular subtypes, among other things, on the presence of the estrogen receptor, progesterone receptor (PR) and human epidermal growth factor receptor 2 (HER2) [93]. Based on their expression, four distinct types of BC can be distinguished: luminal A (ER+ and/or PR+, HER2-), luminal B (ER+ and/or PR+, HER2+/-), HER2-positive (ER-, PR-, HER2+) and TNBC (ER-, PR-, HER2-) [93,94]. The function of PR, analogous to that of ER, is to regulate gene transcription in response to ligand binding [95]. Consequently, its expression represents a significant factor in the pathogenesis of BC. Furthermore, the incidence of luminal type A breast cancer increases with age, thus emphasising the role of sex hormones in the development of BC [96]. In the case of male breast cancer (MBC), an increase in incidence relative to age can also be observed, but the cause of this phenomenon is different. In more than 40% of cases, the development of MBC is attributable to genetic mutations in the *BRCA1*/*BRCA2* or *CHEK2* genes. The majority of cases of MBC are invasive cancers [97].

This has been shown to result in a poorer prognosis, a lower 5-year survival rate and an increased risk of death when compared to the incidence of BC among the female population [98]. Risk factors for the development of MBC have been identified as including Klinefelter syndrome, liver disease or testicular abnormalities, in addition to environmental exposure resulting in an elevated oestrogen-to-androgen ratio [99]. In the context of MBC, the incidence of oestrogen markers has been shown to be comparable to that observed in the population of postmenopausal women [97]. It has been established that the vast majority of MBC cases, approximately 99%, are *ESR1*-positive [100]. As we mentioned estrogen receptor expression represents a core method of BC classification [101]. The presence of ERα has been documented in over 70% of BC cases in women population [102]. The prognosis for BC with ERα is more favourable than for BC without ERα [103]. In normal breast tissue, the gene encoding ERα is expressed selectively in the epithelial compartment, where the receptor is detected in only about 10% of cells. The level of expression in normal cells varies according to factors such as puberty, pregnancy and lactation. In the course of BC, ERα expression is higher than in normal cells and remains at a constant level [104]. In addition to overexpression, increased activity of this receptor may result from its post-translational modifications. This is particularly important in the context of the ligand-independent activation mechanism. An example of such dependencies is the increase in ERα activity with phosphorylated serine residues (S118, S167, S305) and tyrosine (Tyr537) [105]. The isoforms ER46α and ER36α have also been detected in BC cells. ER36α is a particularly important isoform in breast cancer because it is expressed in both ER-positive and ER-negative tumours and has been positively correlated with tumour size, stage and metastasis [106,107]. Moreover, evidence suggests that this isoform may play a role in the development of resistance to tamoxifen treatment by activating sphingosine kinase 1 (SphK1) [107]. *ESR1* gene mutations have also been demonstrated to play a meaningful role in the process of carcinogenesis and BC progression. Research has shown that the majority of the numerous mutations discovered relate to the LBD region. These mutations emerged as a consequence of the response to anti-estrogenic therapy [108].

In triple-negative breast cancer, the ERβ receptor is present in 20–30% of cases, which renders it a promising therapeutic target in this aggressive subtype of breast cancer. However, there is controversy regarding its potential use in TNBC therapy. This controversy pertains to a number of aspects, including the relative inactivity of ERβ in this particular type of cancer, attributable to the low expression of AP1 (activating factor 1, a heterodimer of Fos and Jun proteins) and NF-κB [109]. Notwithstanding the extensive research conducted on the subject, a plethora of inconsistencies persist with regard to the function of ERβ in BC. These outcomes may arise from the presence of various isoforms of the receptor and their distinct capacity to influence clinical trial results [110].

In a manner analogous to ERβ, GPER has been detected in both ER+ and ER- breast cancers [105,111]. As with ERβ, there is a lack of consensus regarding the effect of GPER on the development and progression of BC. A number of studies have demonstrated a correlation between the presence of GPER and poor prognosis [111,112], while others have confirmed its suppressive effect on breast cancer [113]. However, research on BC has revealed elevated GPER levels, which have also been found to impact CAFs, thereby exacerbating tumour proliferation and angiogenesis [114]. The involvement of this receptor in the feedback loop linking the induction of IL-1β in CAFs and the expression of *IL1R1* (interleukin 1 type I receptor gene) induced by hypoxia in TNBC cells has also been verified [115]. The observed variation in results may be attributable to the differential expression of GPER, which has been detected in two distinct locations within cells: the cytoplasm and the cell nucleus. Cytoplasmic expression has been associated with low-stage histological subtypes of cancer, while nuclear expression has been linked to poor differentiation, which is often indicative of malignancy [116]. A similar conclusion was drawn in a study conducted by Martin et al., where low GPER expression in the cytoplasm was found to be associated with an increased mortality rate in breast cancer patients undergoing endocrine therapy [117].

### 5.3. Thyroid Cancer

In 2022, thyroid cancer (TC) was one of the most prevalent cancers among adolescents [118]. Statistics show that proliferative thyroid diseases are more common in women than in men. The preponderance of TC in women is notable, with an incidence of this type of cancer that is three times higher than in men [119]. In view of the marked differences in the incidence of TC according to gender, research was initiated into the impact of hormones on the development of this particular type of cancer [120]. Once again, sex hormones play an important role, this time in the development of thyroid cancer. Estrogen is a potent growth factor for both benign and malignant thyroid cells, which may explain the gender differences in the incidence of thyroid nodules and thyroid cancer. Consequently, it was determined that both normal thyroid tissue and cancerous tissue exhibit sensitivity to changes in estrogen levels [121]. Normal thyroid tissue expresses ERβ at a higher level than ERα [122]. The presence of both ERα and ERβ has been identified in TC cells. However, in TC, the number of estrogen receptor α increases, while the number of ERβ decreases [120]. It has been demonstrated that this dysregulation may result from DNA methylation of the ERβ 5′-untranslated region [123]. Moreover, a positive effect of ERα on tumour development and a protective effect of ERβ have been observed in TC, as well as in many other types of cancer [124].

In the context of papillary thyroid carcinoma (PTC), estrogen receptor signalling, activated by estradiol, exerts a notable influence on the pathogenesis of the disease. These changes include an increased level of vimentin and MMP9 and a concomitant decrease in E-cadherin [125]. In this type of cancer, it has been observed that an increase in the ERα: ERβ expression ratio induces an increase in MMP2 expression, modulating cancer invasion [124]. Research indicates that ERα exerts a regulatory influence on ETV5 (ETS-related molecule) expression, which functions as a mediator in phosphatidylinositol-4,5-bisphosphate 3-kinase catalytic subunit alpha (PI3KCA) activation, thereby contributing to enhanced proliferation and metastasis in TC [126].

However, the results of the studies are not clear. Some of these studies contradict the well-established positive correlation between increased ER expression and the development of PTC. Nevertheless, the role of this receptor in the carcinogenesis and progression of PTC remains unquestioned [127]. Furthermore, the presence of variations in results can be attributed to differences in the methods of receptor detection and the reagents utilised in the immunohistochemical method [128]. It is important to note that a comparable pattern is evident in the case of GPER. While the relationship between GPER levels and TC progression remains unclear, a majority of studies suggest a positive impact on the development of the disease [129]. An alternative position is posited by the analysis conducted by Bertoni’s group, which indicates a correlation between reduced GPER expression and increased lymph node metastasis in primary PTC [130]. The etiology of TC also includes genetic factors that are not associated with ER. These include mutations in the *BRAF*, *RAS* and *RET*/*PTC* genes, which lead to the activation of the oncogenic MAPK pathway [131]. In addition, the risk of TC may also increase in patients with breast cancer who harbour a *CHEK2* mutation. Moreover, radiotherapy for the primary cancer in children also plays a significant role in the development of TC [132]. In addition to heavy metals, radiation and air pollution, obesity is an important environmental risk factor for thyroid cancer. In addition to other factors, the increase in thyroid-stimulating hormone (TSH) levels has been observed to result in the enhancement of thyroid cell proliferation and genetic mutations, which have been identified as significant contributors to the development of TC. In addition, obesity-induced insulin resistance is associated with an increase in insulin-like growth factor 1 (IGF-1), which leads to the activation of AKT/mTOR/PI3K or ERK/RAS/MAPK pathways promoting the development of TC. Furthermore, disturbances in adipokine levels, such as those involving adiponectin, resistin or leptin [133], and increases in insulin concentration induced by insulin resistance [134], may play an important role in the development of TC.

### 5.4. Colorectal Cancer

It is reported that colorectal cancer is responsible for more than half a million deaths per year [56]. Colorectal cancer is unique in its dependence on sex hormones. Studies clearly indicate that the incidence and prognosis of CRC differ depending on gender. A review of the literature on CRC cases has demonstrated sexual dimorphism with regard to incidence, mortality and tumour type [135]. As the statistics show, CRC has been shown to have a higher incidence and mortality rate among men than among women [136]. Furthermore, it has been determined that estrogens play a key protective role in CRC among the female population [135]. Pre-menopausal women demonstrating low ERα and AR levels, in conjunction with high ERβ concentrations, are observed to be the least predisposed to developing CRC [137]. Consequently, a more thorough examination of the receptors stimulated by these substances is warranted. It is well known that estrogen receptors occur naturally in the colonic epithelium. As with the aforementioned types of cancer, in CRC ERα expression increases, while ERβ decreases in relation to the amount in normal cells [138]. The quantitative ratio is reversed in comparison with normal tissue, where the predominant form of ER is the β form [139]. These changes have been demonstrated to have a substantial impact on the deterioration of the patient’s condition and the prognosis, as a result of the roles played by both forms of the receptor in CRC [138]. This assertion is corroborated by a study conducted by Topi et al., which demonstrated the impact of increased ERβ expression and decreased ERα expression on the improvement in overall survival and disease-free survival in CRC patients of both sexes [140]. The same team confirmed the involvement of ERα in metastasis and poorer prognosis in patients with its high expression, among other things through its correlation with β-catenin and CysLT1R (cysteinyl leukotriene receptor 1) [141]. Furthermore, studies conducted by Hases et al. on a mouse model established that the protective mechanism of ERβ in CRC consists in its direct binding to NF-κB activators, thereby inhibiting their activity while stimulating the expression of the NF-κB inhibitor [142]. This finding aligns with the team’s other studies, which suggest that the protective function of ERβ in CRC is based on the inhibition of NF-κB-dependent inflammatory signalling [143]. Additionally, research on ERβ in CRC has revealed a particularly high proportion of ERβ2 and ERβ5 isoforms in samples taken from patients, but their role in this type of cancer remains unclear [144]. The role of GPER in CRC remains to be fully elucidated. However, experimental analyses and clinical cases have produced contradictory results when attempting to determine whether GPER has pro- or anti-cancer properties [145,146]. A study by Abancens et al. provided confirmatory evidence for the suppressor effect of GPER, demonstrating its inhibitory effect on the JUN oncogene and the Wnt/β-catenin pathway, which is overactive in CRC [147]. The primary factors contributing to the development and progression of CRC are the accumulation of mutations in the Wnt signalling pathways, epidermal growth factor receptor (EGFR), and transforming growth factor beta (TGF-β) [148]. In addition to recognised risk factors, such as the use of stimulants and family history, diabetes has been shown to have a significant impact on CRC. It has been demonstrated that this can increase the risk of precancerous polyps by 50%, with a significant effect on the incidence of CRC, particularly among younger demographics [149]. The presence of obesity has been demonstrated to be associated with an elevated risk of colorectal cancer. Research findings have indicated that a one-unit rise in body mass index (BMI) is associated with a 2–3% elevated risk of colorectal cancer [150]. Table 1 provides a summary of the role of the different types of ER in the cancers discussed.

In general, the mechanisms associated with oestrogen receptor complex signalling are extensive and appear to be specific to a given cell type. They can also interact with various signal transduction pathways in cells. In many cases, this signalling can lead to neoplasia or promote malignant transformation. It is now known that oestrogen-dependent cancers are not limited to breast or ovarian cancer. The relationship between cancers occurring with varying frequencies depending on gender is largely related to hormone-dependent regulation. A detailed understanding of this allows for the correct diagnosis of patients and appropriate treatment or preventive measures.

## 6. SERMs/SERDs/AIs

Hormonal/endocrine therapy inhibits the proliferation of cancer cells through the blocking of hormone effects (use of hormone receptor antagonists or downregulation/degradation of hormone receptors) or by inhibiting hormone production [151].

Hormonal therapy is used to treat hormone-dependent cancers such as breast cancer [152], prostate cancer [153], and gynecological cancers [154], but also in the treatment of thyroid cancer [155]. In many hormone-dependent cancers, the functioning of signaling pathways mediated by estrogen receptors, among others, is disrupted [156].

Selective estrogen receptor modulators and selective estrogen receptor degraders/downregulators are used in hormonal therapy estrogen receptor-positive cancer. SERMs block/inhibit signal transmission involving ER (alpha and/or beta). Tamoxifen, toremifene, and raloxifene are selective estrogen receptor modulators currently in use [157,158,159]. Fulvestrant is selective ER downregulator/degrader (SERDs) which bind to ERα, leading to receptor downregulation/degradation and in consequence inhibition of signaling involving the ERα [160]. In addition, hormone therapy for ER-positive cancers uses aromatase inhibitors, enzyme responsible for the synthesis of estrogens from androgens. Third-generation aromatase inhibitors include letrozole, anastrozole, and exemestane [161].

Tamoxifen is one of the most commonly used drugs in adjuvant hormone therapy for ER-positive breast cancer [162]. Tamoxifen’s metabolites, afimoxifen (4-hydroxytamoxifen, 4-OHT) and endoxifen (4-hydroxy, N-desmethyl-tamoxifen) [163,164,165], a nonsteroidal ER antagonist, competitively bind to ER, causing a conformational change that promotes the release of HSP90 and ER dimerization. In the cell nucleus, the ER dimer participates in the activation of AF1 domain and the inhibition of AF2 domain, then binds to ERE in the promoter regions and stimulates AF-1-mediated gene transcription. Transcription of estrogen-responsive genes is inhibited by the tamoxifen-ER dimer because the ligand-dependent AF2 domain is inactivated and the binding of ER coactivators is restricted [166,167].

The antiestrogenic effect of tamoxifen is also achieved through the recruitment of co-repressor proteins, such as NcoR/SMRT (nuclear receptor co-repressor/ silencing mediator of retinoic acid and thyroid hormone receptors) by tamoxifen-ER dimers complex HDAC (histone deacetylases) and HDACs (HDAC3), which leads to the repression of ER-dependent gene transcription [168,169,170,171]. Tamoxifen is an estrogen antagonist in breast epithelium and is therefore used in adjuvant treatment of breast cancer. However, it acts as an agonist in other estrogen-dependent organs, including the endometrium, where it may promote endometrial proliferation disorders, including hyperplasia, and increase the risk of uterine and endometrial cancer [172].

Toremifene, a chlorinated derivative of tamoxifen, is a nonsteroidal SERM used to treat both early and late stages of metastatic ER-positive breast cancer [173]. The main active metabolite of toremifene is 4OH-NDM (N-desmethyl) -toremifene and 4-hydroxy (4-OH) toremifene [174,175] exhibit antagonistic effects to estrogens in target tissues such as breast epithelium, and partial agonistic effects in uterine and bone tissues [173,176]. The mechanism of action of toremifene is analogous to that of tamoxifen. After binding to ER, toremifene metabolites initiate ER dimerization, and the ER-toremifene complex is translocated to the nucleus, leading to activation of the AF1 domain, inactivation of the AF2 domain, and binding of the dimer to the ERE of target genes promoters. The toremifene-ER complex reduces the binding of ER coactivators, except for inactivation AF2 domain, resulting in reduced transcription of estrogens-dependent genes [167,176,177].

Raloxifene is a selective estrogen receptor modulator belonging to the benzothiophenes. Raloxifene is the only benzothiophene SERM used to reduce the risk of breast cancer. Like tamoxifen and toremifene, raloxifene also exhibits agonistic or antagonistic effects on estrogen depending on the target tissue [175,178].

Raloxifene exhibits greater affinity for ER than its main glucuronide conjugate metabolite, raloxifene-4′-β-glucuronide [179,180]. The estrogenic effect of raloxifene occurs in bone and lipid metabolism, while the antiestrogenic effect occurs in breast tissue, and a neutral effect is observed in the endometrium [181,182].

After binding to ER via the benzothiophene ring, raloxifene causes a change in the conformation of ER, which leads to the activation of the AF1 domain and inactivation of the AF2 domain of ER. The raloxifene-ER complex then binds to the ERE of target gene promoters [180]. In addition, the raloxifene-ER complex reduces the binding of ER coactivators, leading to a decrease in the transcription of estrogen-dependent genes. Furthermore, the raloxifene-ER complex recruits co-regulators to further enhance the antiestrogenic effects of raloxifene [167]. However, raloxifene has a different effect on other tissues, such as bone [183]. The raloxifene-ER complex, with the participation of various proteins (adaptors, enhancers), is able to bind to and activate a specific DNA sequence known as the Raloxifene Responding Element (RRE). The binding of the raloxifene-ER complex to RRE causes the transcription of genes involved in the synthesis of specific cellular proteins responsible for the estrogenic growth and proliferative effects of raloxifene on these tissues [184,185].

Fulvestrant is a 7α-alkylsulfinyl analogue of estradiol that competes with estrogens for binding to ER [186]. The major metabolite of fulvestrant is fulvestrant-3-sulfate conjugate [187,188].

Unlike SERMs, which exert both agonist and antagonist effects depending on the target tissue, fulvestrant exhibits exclusively antiestrogenic activity. Fulvestrant exerts its antiestrogenic effects through various mechanisms, including impairment of ER dimerization, inhibition of ER activity, and accelerated ER proteasomal degradation The antiestrogenic effect is the result of competitive binding of fulvestrant to ER monomers, which significantly impairs ER dimerization, fulvestrant-ER complex formation and translocation to the nucleus, resulting in inhibition of transcriptional activity (associated with both the AF1 and AF2 domains) of estrogen-dependent genes [189].The fulvestrant-ER complex is unstable, which may make it to accelerated proteasomal degradation of the ER compared with ER bound to estradiol or SERMs. Accumulation of the fulvestrant-ER complex in the cytoplasm may also promote its degradation, which may reduce the number of functional ERs in cells. The reduction in ER protein levels in cells caused by fulvestrant is not due to a reduction in ER mRNA levels but rather to accelerated receptor degradation due to the instability of the fulvestrant-ER complex [190,191].

AIs are drugs that lower estrogen levels by deactivating aromatase, blocking aromatization, and inhibiting estrogen synthesis [192]. Anastrozole and letrozole are nonsteroidal aromatase inhibitors, containing a triazole functional group that competitively interacts with the heme prosthetic group of aromatase, relative to androgenic substrates. These drugs can be displaced from aromatase binding sites, allowing potential recovery of enzyme activity. Exemestane is a steroidal aromatase inhibitor, which irreversibly inactivates the enzyme by forming covalent bonds with its active site [193,194]. The origin, mechanism of action, and reversibility/irreversibility of aromatase inhibitors influence not only the potency and selectivity of the inhibitors but also their side effect profiles, which are important factors in their clinical use [195].

## 7. Hormone Therapy Resistance

Sex hormones are ubiquitous. Estrogens are present and locally produced in many cancers. Estrogen receptors are not without importance in oncogenesis. Controlling the signalling of hormone receptors, including the oestrogen receptor (ER), inhibits the activity of cancer cells. This strategy forms the basis of hormone therapy for cancer. However, due to the complexity of sex hormone action—including interactions with the receptors themselves, induction of signal transduction through various signalling pathways, and interaction with the tumour microenvironment—a number of processes can lead to resistance to hormone therapy. Genetic, epigenetic and transcriptomic changes in cancer cells affect not only the heterogeneity of cancers, but also the selection and success of anticancer therapies [196,197,198]. Furthermore, cancers consist not only of cancer cells, but also of other cells and molecules in the surrounding TME that influence disease progression and the success of anticancer therapies [199].

The tumour microenvironment—a complex and dynamic structure comprising tumour cells, stromal components, the vascular system and infiltrating immune cells. The TME also plays an important role not only in tumour progression and evasion of immune response, but also in the acquisition of resistance to both conventional and targeted therapies by cancer cells [200,201,202].

Extracellular vesicles, including exosomes, play a key role in modulating complex interactions between cancer cells and the TME, tumour growth, angiogenesis and metastasis. Some components of exosomes, such as signalling proteins, miRNA, and lncRNA [203,204], may contribute to the development of resistance to treatment, including hormone therapy [205,206].

During anticancer hormone therapy, which brings significant benefits, intrinsic (primary, de novo) resistance or acquired resistance may occur [207]. Primary resistance is defined as disease progression within 6 months of starting hormonal treatment for metastatic breast cancer or recurrence within 2 years of starting adjuvant therapy for early breast cancer. Secondary hormone resistance, defined as progression occurring 6 or more months after the start of hormone therapy for metastatic breast cancer, ultimately develops in most patients. Recurrence during adjuvant hormone therapy, after the first 2 years or within 1 year of completion of adjuvant hormone therapy, is also commonly characterised as acquired secondary resistance [208,209].

Tamoxifen is often used in the adjuvant treatment of ER-positive breast cancer [210]. Despite the benefits of tamoxifen in patients with all stages of ERα+ positive breast cancer, drug resistance may develop [211]. Resistance to tamoxifen treatment may be caused by disruption of multiple molecular pathways: ER signalling, tyrosine kinase receptor signalling pathways (HER2, EGFR, fibroblast growth factor receptor (FGFR) and insulin-like growth factor 1 receptor (IGF1R)), the PI3K-PTEN/AKT/mTOR pathway, and NF-κB signalling [212,213,214,215].

Additional mechanisms of tamoxifen resistance include an imbalance between anabolism and catabolism of estradiol [216], altered bioavailability of tamoxifen [217], increased angiogenesis, heterogeneity of tumour cell populations, or overexpression of growth factors [218,219].

Growth factors that activate tyrosine kinases trigger signalling pathways activated by receptor tyrosine kinases (RTKs), and the non-genomic activity of cell membrane-associated estrogen receptors facilitates cross-communication between these receptors and signalling pathways [213]. Overexpression and activation of EGFR and HER2 leads to cell proliferation and survival through activation of MAPK and PI3K/AKT signalling pathways, thereby contributing to the development of resistance to endocrine therapy [220,221].

Possible causes of tamoxifen resistance associated with ER include increased signalling of growth factors and ER associated with the cell membrane [213], loss of ERα expression and acquired ERα mutations [222]. Loss of ERα expression may be the main mechanism of de novo resistance to hormone therapy and is mainly associated with epigenetic changes i.e. abnormal methylation of CpG islands and increased histone deacetylation [223,224]. ERα mutations can lead to the formation of a receptor with altered properties, which may result in altered molecular signalling. The G protein-coupled ER may also play a role in tamoxifen resistance. Signalling by GPER occurs through transactivation of EGFR and involves tyrosine kinases from the Src, MAPK/ERK and CREB/CRE families [225,226,227].

In women, adrenal and ovarian androgens are the source of pre- and post-menopausal estrogens, as they are converted into estradiol [228]. The androgen receptor (AR) is a ligand (androgen)-activated transcription factor and a member of the nuclear receptor (NR) superfamily. Tumours resistant to tamoxifen show higher levels of AR expression than tamoxifen-sensitive tumours. High AR expression may have an adverse effect on the prognosis of tamoxifen treatment for ERα-positive breast cancer, as increased AR expression may potentially enhance the agonistic properties of tamoxifen [229,230]. The ability of AR to regulate gene transcription results from its interaction with specific DNA sequences located near or within the promoter of the target gene [231]. This is a classic ligand-dependent genomic mechanism [232]. A ligand-independent mechanism triggers AR activation through signalling pathways that include JAK/STAT3, MAPK, NOTCH, and PI3K/mTOR/AKT [233,234]. AR can also rapidly initiate cytoplasmic signalling cascades, including activation of protein kinase A, protein kinase C and ERK, through a non-genomic mechanism involving binding to cytoplasmic and membrane-associated proteins such as c-Src [235].

A new molecular target in the potential treatment of tamoxifen-resistant breast cancer is the Hedgehog signalling pathway, along with the proteins patched 1 and patched 2 (PTCH1 and PTCH2), Hedgehog-interacting protein HHIP and Glioma-associated oncogene homolog 1 and 2 (GLI1/GLI2), which can trigger positive or negative feedback in this pathway [236]. Additionally, a pathway that may interact with Hedgehog signalling in breast cancer is TGF-β, which stimulates the upregulation of GLI1 and GLI2 transcription [237,238]. The PI3K/AKT pathway is also involved in the activation of Hedgehog signalling and may play a role in promoting resistance to tamoxifen [239].

Therapies targeting various receptors and individual components of signalling pathways are currently used not only to improve treatment efficacy but also to prevent the development of resistance to standard treatment. CDK4/6 inhibitors are drugs used to treat certain types of HR+/HER2- breast cancer by blocking the cyclin-dependent kinases 4 and 6 (CDK4/6) enzymes, which are crucial for cell division. Palbociclib and abemaciclib are both CDK4/6i used to treat hormone receptor-positive (HR+), HER2-negative breast cancer, often in combination with endocrine therapy. However, despite the enormous improvement achieved with the introduction of CDK4/6i in combination with hormone therapy, 10% of patients do not respond to treatment, developing de novo resistance. Conversely, others may develop acquired resistance to CDK4/6 inhibitors and disease recurrence [240,241].

Therefore, inhibitors of other important targets with potential involvement in the development of resistance, such as: mTOR inhibitors (Everolimus) [242], PI3K inhibitors (Alpelisib, Pictilib and Buparlisib) [243], AR antagonists (Enzalutamide and Bicalutamide) [244,245], histone deacetylase inhibitors (Entinostat and Vorinostat) [246,247], and MAPK pathway inhibitors (Selumetinib and Ralimetinib) [248,249,250] have been progressed, and some of them are already in clinical use.

A new generation of anti-estrogen therapies has been designed to overcome some of these resistance mechanisms and eliminate the limitations of currently available hormone therapy, e.g., the agonistic activity of tamoxifen. These therapies include variations on existing drug classes, including SERMs other than tamoxifen and new orally administered SERDs. New-generation SERDs reduce ER levels through proteasome-dependent degradation [251].

New classes of anti-estrogen drugs include complete estrogen receptor antagonists (CERANs), selective covalent estrogen receptor antagonists (SERCAs), and proteolysis-targeting chimeras (PROTACs) targeting ER. These drugs are at various stages of development and are being evaluated in both early and metastatic stages of cancer.

SERMs exhibit ER antagonist or agonist activity, depending on the cell type, through the recruitment of different coactivators and correpressors. SERMs inhibit the AF2 of ER but allow agonistic signalling through the AF1 via other signalling pathways such as PI3K-AKT-mTOR, RAS-RAF-MAPK and CDK4/6-RB-E2F. Activation of some of these pathways may be involved not only in primary or secondary resistance to hormonal therapy but also to CDK4/6i [252].

Lasofoxifene is a new-generation non-steroidal SERM with ER binding capacity comparable to 17β-estradiol. Clinical trials are evaluating the efficacy of lasofoxifene in the treatment of metastatic breast cancer in women with *ESR1* mutation (compared to fulvestrant) who have progressed after prior treatment with AIs and CDK4/6i [253], and the efficacy of combining lasofoxifene with abemaciclib (a CDK4/6 inhibitor) [254].

SERDs not only act as competitive ER antagonists, they also induce proteasome-dependent degradation of the ER [255]. Fulvestrant is a SERD administered intramuscularly, which is a certain limitation. Currently, orally administered SERDs are being evaluated in clinical trials for use in metastatic, adjuvant and neoadjuvant settings. Elacestrant (RAD1901) is an orally bioavailable SERD, which is a dose-dependent mixed ER agonist/antagonist. Elacestrant at high doses acts as a direct ER antagonist as well as selective downregulator of ER. The binding of RAD1901 allows the presentation of a protein–protein interaction surface in the hinge region of the DNA and hormone-binding domain of ERα/*ESR1*, crucial for transcriptional activity. Moreover, increased receptor occupancy results in the receptor being targeted for degradation, which quantitatively inhibits ERα/*ESR1* signalling [256,257].

Elacestrant has been approved for the treatment of advanced or metastatic breast cancer in patients with ER+, *ESR1*-mutated and HER2-negative (HER2-) profiles, after progression following at least one line of endocrine therapy [258]. Elacestrant in combination with abemaciclib in patients with brain metastasis from ER+, HER2-negative breast cancer is also being investigated [259].

Other orally administered SERDs include: giredestrant (GDC-9545), also used in combination with palbociclib, everolimus and in combination with CDK 4/6 inhibitors, ipatasertib, inavolisib, everolimus and samuraciclib [207]; camizestrant (AZD9833) used as monotherapy and in combination with palbociclib and in combination with abemaciclib, everolimus and capiwasertib [260] and imlunestrant (LY-3484356). Imlunestrant is an oral SERD, and synergistic effects have been observed in combination with abemaciclib, everolimus, and alpelisib [261]. SERDs include Rintodestrant (G1T48), AZD9496, ZN-c5, D-0502 (Taragarestrant) and Borestrant (ZB-716). Rintodestrant is an oral SERD that competitively binds and degrades the estrogen receptor. AZD9496 is an oral, non-steroidal, selective ER degrader and inhibitor of ER-dependent signalling pathways. Borestrant is fulvestrant modified with boronic acid, with oral bioavailability [22].

Proteolysis targeting chimerics are dual-function hybrids that simultaneously bind to a specific target protein, such as ER, and an E3 ubiquitin ligase, causing ubiquitination and degradation of the target ER protein via the ubiquitin-proteasome system [262]. Because their mechanism of action is catalytic, they are able to promote protein degradation even at low doses. The most advanced PROTAC is ARV-471 [263,264].

Complete estrogen receptor antagonists block both the AF1 and AF2 transcription activation domains of ER. The AF1 domain can be activated by signalling pathways such as mTOR, PI3K and MAPK, and the AF2 domain, which is activated by the estrogen ligand itself. Activation of AF1 and AF2 leads to gene transcription and cell proliferation. CERANs directly inhibit AF2, allowing agonistic signalling involving AF1 via other signalling pathways [265]. OP-1250 is an orally bioavailable CERAN that also acts as a SERD inducing ER degradation. OP-1250 (Palazestrant), is a complete estrogen receptor antagonist, inhibits wild-type and mutant ER-positive breast cancer models as monotherapy and in combination with other drugs [266].

The first-in-class selective covalent estrogen receptor antagonist drug, H3B-5942, covalently inactivates both wild-type and mutant ERα by targeting Cys530 and forcing a unique antagonistic conformation. H3B-5942 potently suppresses ERα function and demonstrates potent antiproliferative and antitumour activity in monotherapy and in combination with CDK4/6 inhibitors (palbociclib, ribociclib, and abemaciclib) or mTOR inhibitors (everolimus, temsirolimus, sirolimus), leading to synergistic activity [267].

Moreover, microRNAs (miRNAs) have emerged as modulators of therapy response and resistance to hormonal therapy [268]. lncRNAs (long non-coding RNAs) also play a leading role in the initiation, progression and resistance to anti-estrogens [269].

Understanding the mechanisms that lead to resistance to hormone therapy enables us to avoid ineffective therapies and propose strategies to circumvent this resistance, providing direction for the search for new drugs.

## 8. Summary

Hormones are one of the most important groups of substances that regulate how our body functions. At the same time, some cancers use hormones to control their growth and spread. Understanding the mechanisms of action and connections in hormonal regulation is therefore an important diagnostic and therapeutic tool. The wide range of interactions between hormone-dependent cancers and their microenvironment is a promising area of research for the development of new therapies. In addition to the pharmaceutical treatments, hormonal therapy for cancer employs techniques that involve the removal of sex hormones from the body. One such technique is castration, which primarily inhibits the development of breast and endometrial cancer in women, as well as prostate cancer in men. The main castration methods include surgical castration and pharmacological castration, which does not require the removal of the gonads. Pharmacological castration is a therapeutic intervention that involves the administration of gonadotropin-releasing hormone (GnRH) analogues. These pharmaceuticals are designed to bind to the receptor located within the pituitary gland, thereby inducing a castration effect. This therapeutic modality is predominantly employed in the management of premenopausal women diagnosed with breast cancer, particularly those below the age of 35. In male patients, this method is utilised when surgical castration is contraindicated or when estrogen therapy is not possible [270]. However, it should be noted that this treatment method is not without its potential disadvantages. In women diagnosed with breast cancer, the induction of premature menopause through the administration of GnRH agonists/antagonists has been proven to be both effective and reversible. However, this treatment is associated with a considerable deterioration in quality of life and a long-term risk of mortality [271]. This method represents the primary treatment option for metastatic prostate cancer (PC). However, it has often been observed that this results in cancer cells becoming insensitive to androgens, which in turn leads to the development of castration-resistant PC (CRPC) with a poor prognosis [272]. In addition to their role in oncological treatment, hormone therapies also play an important role in other aspects of life. Hormone replacement therapy (HRT) involves the administration of agents that induce an estrogenic or progestogenic effect. It is indicated in cases where the ovaries cease to produce these hormones naturally, as a result of cancer treatment, as well as genetic and autoimmune diseases, cardiovascular diseases and accelerated bone loss [273]. However, the potential for developing hormone-dependent cancer should not be overlooked. The type of HRT used should therefore be appropriately selected according to the patient’s age and health status in order to minimise this risk [274]. The risk of developing cancer is also influenced by the use of contraceptives. Research has indicated a correlation between the ingestion of oral contraceptives and an elevated risk of BC in women, particularly those with a mutation in the *BRCA1* or *BRCA2* gene, in comparison with women who do not utilise such contraceptives. This elevated risk persists for a more than 10-year period following the end of use [275]. However, in cases of OC risk in these patients, oral contraceptive pills play a chemopreventive role. This enduring effect persists for a period of up to 15 years following the discontinuation of the drug. It is estimated that the use of oral contraceptive pills reduces the risk of OC by more than 42%. This duality of action necessitates a meticulous analysis of each case by doctors, with a view to selecting the most appropriate type of substance [276].

However, it should be noted that exogenous estrogens are not only associated with therapeutic use. Humans are constantly exposed to the effects of these substances, which are present in the environment. Xenoestrogens are synthetic substances found in plastics, preservatives, pesticides, and other such products, which have an ability to bind to the ER and to activate or block its activity. For instance, bisphenol A has been demonstrated to disrupt ER activity, thereby affecting intracellular signalling and inducing an increase in ER expression. This results in increased cell proliferation, an increase in the number of cells with ER and PR, and activation of AKT and ERK pathways, which in turn leads to, among other things, an increased risk of BC, PC and bladder cancer [277].

In addition to xenoestrogens, exposure to phytoestrogens is a constant factor in the human environment. Unlike xenoestrogens, these compounds occur naturally in plants and share a chemical structure with mammalian estrogens. This enables them to exert a regulatory influence over cellular processes via ER. These properties have led to their widespread use as a treatment for menopausal symptoms without resulting in side effects. However, the ability of these compounds to modulate signalling pathways through ER activation may contribute to the development of tumours. For instance, genistein has been observed to increase the expression of ERα, AKT and NF-κB, thereby stimulating cell proliferation. However, it has been demonstrated that certain phytoestrogenic compounds possess anti-cancer properties. As demonstrated in the relevant studies, the stereospecificity of phytoestrogens plays a pivotal role in their biological activity [278]. The role of plants in providing compounds with hormonal activity extends beyond this. They have the ability to absorb estrogenic steroids present in water and soil, subsequently accumulating these chemicals within their own tissues. The consumption of these plants has been demonstrated to exert a substantial influence on estrogen levels within the human body [279]. 

In light of the multifaceted mechanisms of estrogen receptor ER activation exhibited in this article, the extensive cellular effects they engender, and the numerous sources of exposure to substances that interact with them, it is crucial to emphasise the pivotal role of ER in developing novel therapeutic interventions that target hormone-dependent cancers.

## Figures and Tables

**Table 1:** A comparison of the role of different types of ER in selected cancers.

	Estrogen Receptor Expression	Estrogen Receptor Level Compared to Normal Tissue	Poor Prognosis Connection	References
Ovarian cancer	ERα+ ERβ+ GPER+	ERα ˜ Erβ↓ GPER ˜	Erβ↓ ERβ ^ND^ GPER↑/↓	[85,86,87,89,90,91]
Breast cancer	ERα+/- ERβ+/- GPER+	ERα↑ ERβ ^ND^ GPER ^ND^	Erα↓ ERβ ^ND^ GPER↑/↓	[104,105,106,107,109,111,112,113]
Thyroid cancer	ERα+ ERβ+ GPER+	Erα↑ Erβ↓ GPER ^ND^	Erα↑ Erβ↓ GPER↑/↓	[120,124,129,130]
Colorectal cancer	ERα+ ERβ+ GPER+	Erα↑ Erβ↓ GPER ^ND^	Erα↑ Erβ↓ GPER↑/↓	[138,139,140,145,146,147]

+: expression; -: non expression; ↑: increase; ↓: decrease; ˜: similar level; ^ND^: not data avalible; ERα: estrogen receptor alpha; ERβ: estrogen receptor beta; GPER: G protein-coupled estrogen receptor.

## Data Availability

No new data were created or analyzed in this study.

## Abbreviations

The following abbreviations are used in this manuscript:

AF	Activation function domains
Akt	Protein kinase B
AR	Androgen receptor
AIs	Aromatase inhibitors
AP1	Activating factor 1
AR	Aromatase
BC	Breast cancer
BMI	Body mass index
CAFs	Cancer-associated fibroblasts
cAMP	Cyclic adenosine monophosphate
CCL2	CC-chemokine ligand 2
CCNA2	Cyclin A2
CDK4/6	Cyclin-dependent kinases 4 and 6
CDK4/6i	Cyclin-dependent kinase 4/6 inhibitor
CERANs	Complete estrogen receptor antagonists
COX-2	Cyclooxygenase-2
CRC	Colorectal cancer
CREB	Cyclic AMP-responsive element-binding protein
CRPC	Castration-resistant prostate cancer
CSCs	Cancer stem cells
CysLT1R	Cysteinyl leukotriene receptor 1
DBD	DNA-binding domain
ECM	Extracellular matrix
EGF	Epidermal growth factor
EGFR	Epidermal growth factor receptor
EMT	Epithelial–mesenchymal transition
ER	Estrogen receptor
ER+	Estrogen receptor-positive
ERα	Estrogen receptor alpha
ERβ	Estrogen receptor beta
ERE	Estrogen response elements
ERK-1	Extracellular signal-regulated kinase 1
ER-positive	Estrogen receptor-positive
*ESR1*	Estrogen receptor 1 gene
*ESR2*	Estrogen receptor 2 gene
E2	17β-estradiol
FGFR	Fibroblast growth factor receptor
FBLN-1C	Fibulin-1C
*FOXP3*	Forkhead box P3 gene
GLI1	Glioma-associated oncogene homolog 1
GLI2	Glioma-associated oncogene homolog 2
GnRH	Gonadotropin-releasing hormone
GPER	G protein-coupled estrogen receptor
HA	Hyaluronic acid
HAS2	Hyaluronan synthase 2
HDAC3	Histone deacetylase 3
HDAC6	Histone deacetylase 6
HER2	Human epidermal growth factor receptor 2
HGF	Hepatocyte growth factor
HHIP	Hedgehog-interacting protein
HO-1	Heme oxygenase-1
HRT	Hormone replacement therapy
H3K4me3	Histone H3 lysine 4 trimethylation
IGF	Insulin-like growth factor
IGF-1	Insulin-like growth factor 1
IGF1R	Insulin-like growth factor 1 receptor
IL-1β	Interleukin-1 beta
*IL1R1*	Interleukin 1 type I receptor gene
IL-2	Interleukin-2
IL-4	Interleukin-4
IL-6	Interleukin-6
IL-7	Interleukin-7
IL-8	Interleukin-8
IL-11	Interleukin-11
ITGA5	Integrin 5α
LBD	Ligand-binding domain
MAPK	Mitogen-activated protein kinase
MBC	Male breast cancer
MCAM	Melanoma cell adhesion molecule
MDSC	Myeloid-derived suppressor cell
MMPs	Matrix metalloproteinases
MMP2	Matrix metalloproteinase-2
MMP9	Matrix metalloproteinase-9
mTOR	Mammalian target of rapamycin
NF-κB	Nuclear factor kappa-light-chain-enhancer
NKs	Natural killer cells
NR	Nuclear receptor
NTD	N-terminal nucleotide binding domain
OC	Ovarian cancer
PC	Prostate cancer
pCREB1	Phosphorylated cAMP-responsive element-binding protein 1
PD-1	Programmed cell death protein-1
PDGF	Platelet-derived growth factor
PD-L1	Programmed cell death-ligand 1
PIP2	Phosphatidylinositol 4,5-bisphosphate
PIP3	Phosphatidylinositol 3,4,5-trisphosphate
PI3K	Phosphatidylinositol 3-kinase
PI3KCA	Phosphatidylinositol-4,5-bisphosphate 3-kinase catalytic subunit alpha
PR	Progesterone receptor
PROTACs	proteolysis-targeting chimeras
PTC	Papillary thyroid carcinoma
PTCH1	Proteins patched 1
PTCH2	Proteins patched 2
PTEN	Phosphatidylinositol-3,4,5-trisphosphate 3-phosphatase
Ras	GTPase HRas
RRE	Raloxifene responding element
RTKs	Receptor tyrosine kinases RTKs
SERCAs	Selective covalent estrogen receptor antagonists
SERDs	Selective estrogen receptor downregulators
SERMs	Selective estrogen receptor modulators
Src	Proto-oncogene tyrosine-protein kinase
STAT3	Signal transducer and activator of transcription 3
STAT5	Signal transducer and activator of transcription 5
TC	Thyroid cancer
Treg	Regulatory T lymphocyte
TAMs	Tumour-associated macrophages
TANs	Tumour-associated neutrophils
TGF-β	Transforming growth factor beta
TGFβ1	Transforming growth factor beta 1
TIME	Tumor immune microenvironment
TIMPs	Tissue inhibitors of metalloproteinases
TME	Tumour microenvironment
TNBC	Triple-negative breast cancer
TNF-α	Tumour necrosis factor alpha
TSH	Thyroid-stimulating hormone
VEGF	Vascular endothelial growth factor
